# Expression of cellobiose dehydrogenase gene in *Aspergillus niger* C112 and its effect on lignocellulose degrading enzymes

**DOI:** 10.3389/fmicb.2024.1330079

**Published:** 2024-03-18

**Authors:** Yanan Zhong, Zepan Guo, Meiqun Li, Xiaojiang Jia, Baiquan Zeng

**Affiliations:** ^1^College of Life Science and Technology, Central South University of Forestry and Technology, Changsha, China; ^2^Hunan Academy of Forestry, Changsha, China

**Keywords:** cellobiose dehydrogenase, *Aspergillus niger* C112, protoplast transformation, Cellulase, lignocellulose degrading enzyme

## Abstract

Cellobiose dehydrogenase (CDH) is one of the cellulase auxiliary proteins, which is widely used in the field of biomass degradation. However, how to efficiently and cheaply apply it in industrial production still needs further research. *Aspergillus niger* C112 is a significant producer of cellulase and has a relatively complete lignocellulose degradation system, but its CDH activity was only 3.92 U. To obtain a recombinant strain of *A. niger* C112 with high cellulases activity, the CDH from the readily available white-rot fungus *Grifola frondose* had been heterologously expressed in *A. niger* C112, under the control of the gpdA promoter. After cultivation in the medium with alkali-pretreated poplar fiber as substrate, the enzyme activity of recombinant CDH reached 36.63 U/L. Compared with the original *A. niger* C112, the recombinant *A. niger* transformed with *Grifola frondosa* CDH showed stronger lignocellulase activity, the activities of cellulases, *β*-1, 4-glucosidase and manganese peroxidase increased by 28.57, 35.07 and 121.69%, respectively. The result showed that the expression of the *gcdh* gene in *A. niger* C112 could improve the activity of some lignocellulose degrading enzymes. This work provides a theoretical basis for the further application of *gcdh* gene in improving biomass conversion efficiency.

## 1 Introduction

Lignocellulose is one of the most abundant renewable resources in the world with great potential in the development of clean energy and biobased chemicals (Han et al., 2019; Shen and Sun, 2021). It is eco-friendly, does not compete with food, is cheap, carbon-neutral sustainable and abundant, etc. (Dessie et al., 2020). However, it is common that a large number of lignocellulose resources ended with burning, the cheap, fast but polluted treatment. Lignocellulose has a complex composition consisting mainly of cellulose, hemicellulose, and lignin, which are rich in intermolecular linkages bonds (e.g., *β*-1,4 glycosidic bonds, hydrogen bonds) and form a strong cellulose-hemicellulose-lignin network structure that is resistant to erosion, and thus lignocellulose is inefficiently bioconverted (Jiang et al., 2018; Zoghlami and Paës, 2019). Cellulases is a complex enzyme system, including endo-1,4-*β*-D-glucanohydrolase (EG), *β*-1,4-glucosidase (BGL), and *β*-1,4-D-glucan-4-glucanohylases (CBH). Cellulases is vital for bioconversion of lignocellulose, but the activity of natural cellulase is generally low because of carbon metabolism repression (Foreman et al., 2003; Kumar et al., 2008). There are many methods to increase the activity of cellulases, and the cellulases auxiliary protein is one of the resolvents. Cellulases auxiliary proteins are mainly lytic polysaccharide monooxygenase (LPMO), cellobiose dehydrogenase (CDH), expansin, and swollenin (SWOI), which can effectively promote the hydrolysis of cellulose, and some of them have a certain cellulose hydrolysis ability (Chen et al., 2010; Harris et al., 2010; Wang and Lu, 2016; Chase et al., 2020).

CDH (EC 1.1.99.18) belong to the glucose-methanol-choline (GMC) family of the oxidoreductases (Janusz et al., 2017), which is a kind of extracellular enzyme secreted by various white-rot fungi such as *Trametes versicolor*, *Grifola frondose*, *Volvariella volvacea*. *Grifola frondose* is an edible, readily available and safe strain (Wu et al., 2021). CDH consists of two domains, an N-terminal heme b-containing cytochrome domain (CYT) and a dehydrogenase domain-containing flavin adenine dinucleotide (FAD-DH), which are linked by a hydroxyl-rich amino acid linker for exchanging electrons between two domains (Igarashi et al., 2002; Kracher et al., 2016). When the available electron acceptors appeared, such as quinones, chelated Fe^3+^ and Cu^2+^, CDH can catalyze the oxidation of cellobiose, lactose, and cello-oligosaccharides to their corresponding 1,5-lactones (Henriksson et al., 2000; Ludwig and Haltrich, 2002), that can be further hydrolyzed into the carboxylic acids, effectively alleviating the end-product inhibition in the process of cellulose degradation and increasing cellulase activity (Kadek et al., 2015; Du et al., 2018). CDH also can provide electrons for the reduction process of LPMO to boost the action of hydrolytic cellulases (Breslmayr et al., 2020). And some studies found that CDH can promote the degradation of manganese peroxidase (Mn P), which played a role in lignin degradation (Huang et al., 2001). In a word, CDH has great application prospects in the degradation of lignocellulose and cellulose indeed.

Poplar, as a part of the lignocellulose resource, has a wide range of applications. In addition to the application in packaging, building materials, and improving the performance of other materials, it can also be used to produce biofuel, lipid, and so on (Crawford et al., 2016; Samavi et al., 2019). To maximize the value of poplar, improving the efficiency of bioconversion of poplar is the key. In this paper, we constructed CDH genes of *Grifola frondose* expression systems in *Aspergillus niger* C112, studied changes of enzyme activity of lignocellulose degrading enzyme, and preliminary enzymatic properties of recombinant CDH, providing a new idea for the efficient utilization of poplar resources.

## 2 Materials and methods

### 2.1 Microbial strains and vectors

*A. niger* C112 was preserved in Fermentation Engineering Laboratory of Central South University of Forestry and Technology, which was an engineering strain obtained by UV mutagenesis of original strain *A. niger* 30,786. *Grifola frondose* was purchased from Hubei Wuhan Huazhong Edible Fungi Cultivation Research Institute. *Escherichia coli* DH5α and the pUCm-T vector were purchased from Sangon Biotech. Vector pBARGPE1-Hygro was purchased from FENGHUISHENGWU.

### 2.2 Cloning of the *gcdh* gene

*Grifola frondose* was cultivated in PDA liquid medium for 14 days. The culture was incubated at 28°C with rotary shaking of 160 rpm/min. After cultivation, the mycelium was harvested by filtration and then ground to fine powder using a pestle in liquid nitrogen and total DNA was extracted using a genomic DNA kit purchased from TIANGEN, with some modifications.

The amplification primers of *Grifola frondose*-*cdh* were, respectively, GF/GR (Table 1), supplied with the Sma I restriction sites (underlined). These primers were designed according to the sequence of the *Grifola frondose* CDH (GenBank Accession No: AB083245.1). PCR (polymerase chain reaction) was performed with LA Taq DNA polymerase and a deoxynucleoside triphosphate (dNTP) mix from Takara, oligonucleotide primers from General Biol, and a T-100TM thermal cycler from Bio-Rad Laboratories. PCR was done in a reaction of 20 μL containing 2 μL of 10LA PCR Buffer II, 2 μL of dNTP Mixture, 0.2 μL of Takara LA Taq DNA polymerase, 0.2 μM each primer, 1 μL of DNA template, and 13 mL of ddH2O with a cycling protocol as follows: 34 cycles at 95°C for 30 s, 55°C for 30 s, and 72°C for 3 min. The PCR product was, respectively, cloned into the TA cloning vector, pUCm-T, and sequenced by Beijing Genomics institution. Compare and analyze the gene sequencing results of *gcdh* with the published *gcdh* sequences of *Grifola frondosa* on the online website NCBI BLAST.

**Table 1:** The amplification primers of *Grifola frondose*-*cdh.*

Primers	Sequences
GF	5′- ATATCGAATTCCTGCAGCCCGGGTCACGGTCCCCCTGCCAAAG-3′
GR	5′- TCGACTCTAGAGGATCCCCCGGGATGTTCGGACATCTACTGTT-3′

### 2.3 Bioinformatics analysis of gCDH

The amino acid sequence of gCDH was analyzed using ExPASy-ProtParam, the secondary and tertiary structures of gCDH were analyzed using PredictProtein and SWISS-MODEL, respectively, and the structural domains of the protein were analyzed according to SignalP and NCBI. Screening of amino acid sequences of cellobiose dehydrogenase with high similarity from NCBI, perform multiple sequence alignment using Jalview, and analyze the conserved sequences of CDH protein. And construction of phylogenetic tree using MEGA 11 to analyze the evolution of CDHs from 16 sources (Table 2).

**Table 2:** Source of CDH amino acid sequences involved in comparison.

Filamentous fungi	Accession number*	Filamentous fungi	Accession number*
*Trametes versicolor*	AAC50004.1	*Phanerochaete chrysosporium*	AAC49277.1
*Grifola frondose*	BAC20641.1	*Rhizoctonia solani*	CUA69410.1
*Aspergillus nomiae*	XP_015405088.1	*Stachybotrys bisbyi*	ADT70778.1
*Aspergillus luchuensis*	GAT21023.1	*Trametes sanguinea*	AGS09132.1
*Colletotrichum higginsianum*	TIC92379.1	*Trametes cinnabarina*	AAC32197.1
*Diplocarpon rosae*	PBP22038.1	*Lachnellula occidentalis*	TVY48802.1
*Gelatoporia subvermispora*	EMD36613.1	*Lachnellula willkommii*	TVY91786.1
*Humicola insolens*	AAF69005.1	*Auricularia subglabra*	EJD48894.1

*All amino acid sequences were derived from NCBI (Home–Protein–NCBI) (nih.gov).

### 2.4 Construction of expression system

The recombinant plasmid pBARGPE1-Hygro-*gcdh* was constructed by the way of homologous recombination, using EasyGeno Single Assembly Cloning Kit from TIANGEN.

### 2.5 Transformation of *Aspergillus niger* C112

*A. niger* C112 was cultivated in YPD liquid medium for 16 h at 28°C with rotary shaking of 160 rpm/min. After cultivation, the mycelium was harvested by filtration and the mycelium was washed twice with 30 mL of pH 5.5 sterile osmotic stabilizer, centrifuged and the supernatant was removed. The enzyme solution (containing 1.0% snailase, 0.5% cellulose, 0.5% lywallzyme) was added according to solid–liquid ratio of 1:4, incubated for 3 h at 30°C, 160 rpm/min. After incubation, the enzymatic hydrolysate was filtrated by a small mass of cotton. The filtrate was washed twice with 20 mL of pH 7.5 sterile isotonic solution. The protoplasts were collected by centrifugation for 5 min at 4°C, 5000 rpm/min, and resuspended in 100 μL of pH 7.5 sterile isotonic solution.

The transformation was mediated by PEG-4000. 10 μg of the expression vector was added into the protoplast suspension, with 25 μL of pH 7.5 mediated solution, incubated in ice for 20 min. After incubation, 1 mL of pH 7.5 mediated solution and 2 mL of pH 7.5 sterile isotonic solution were added. They were mixed with 10 mL of the top regeneration medium and poured on the bottom regeneration medium plates immediately. The plates were incubated at 28°C until colonies appeared. The composition of the bottom regeneration medium was the same as the top medium, except for 2% agar. The positive transformants were screened using a regeneration medium containing 100 μg/mL hygromycin B, and the regenerated colonies were transferred to the regeneration medium containing 200 μg/mL hygromycin B. The total DNA of positive transformants was extracted and verified by PCR with primers GF/GR. The original strain *A. niger* C112 was used as blank control.

### 2.6 Expression verification of *gcdh*

Selected transformants of *A. niger* were inoculated with 106 spores at 28°C on solid medium. After 6 days of culture, collected mycelium, and total RNA was extracted by fungal total RNA isolation kit from Sangon Biotech. The isolated RNA was used for RT-PCR. RNA template solution containing 1 μL 10 mM dNTP Mix, 1 μL 2.5 μM Oligo dT Primer, 2 μL total RNA, and 6 μL RNase free water was reacted at 65°C for 5 min to denature and anneal the RNA. The reverse transcription system included 10 μL RNA template solution, 4 μL RTase reaction buffer, 0.5 μL 40 U/μL RNase inhibitor, 0.5 μL 200 U/L Evo M-MLV RTase, and 5 μL RNase free water. The reverse transcription reaction condition was 42°C for 30 min, then 95°C for 5 min. All the reverse transcription reagents were purchased from Accurate Biology. The products of RT-PCR were detected by 1% agarose gel electrophoresis.

### 2.7 Enzyme activity detection

*A. niger* C112 and *A. niger* C112-*gcdh* were inoculated with 10^7^ spores at 28°C in 100 mL liquid PDA medium. The seed liquid was cultured for 2 days and inoculated in the fermentation medium. The inoculation amount was 5%. The poplar in the fermentation medium was soaked in 4% sodium hydroxide and treated at 121°C for 90 min. The treated poplar was washed to neutral, dried, and broken (less than 100 mesh). The fermentation medium was incubated at 28°C with rotary shaking of 160 rpm/min. Extracted the supernatant of the fermentation broth and measured the activity of the following enzymes.

CDH activity was determined by spectrophotometric assay (Baminger et al., 1999) performed at 37°C in a 1 mL reaction mixture containing 300 mM cellobiose, 3 mM DCIP, sodium acetate buffer (pH 4.0), and crude enzyme solution. One unit of enzymatic activity of CDH was described as:1 L enzyme solution catalyzes the reduction of 1 μmol of DCIP per minute (U/L).

Filter paper activity (FPA), which was used to measure the overall activity of cellulases, was determined by DNS Colorimetry (Saqib and Whitney, 2011). Definition of enzyme activity: under the condition of 50°C, pH 4.8. One unit of enzymatic activity of FPA was described as: 1 mL cellulases solution catalyzes the production of 1 μg reducing sugar per minute (U/mL).

*β*-1,4-D-glucan-4-glucanohylases (CBH) activity was determined by p-Nitrophenol (Deshpande et al., 1984). The standard curve of p-Nitrophenol was constructed. The enzyme reaction system contained 1 mg/mL p-Nitrophenol-D-cellobioside (PNPC) and diluted sample. After incubation at 50°C for 30 min, added 1 M Na_2_CO_3_ to terminate the reaction. The absorbance value was measured at the wavelength of 410 nm. One unit of enzymatic activity of CBH was described as: 1 mL enzyme solution catalyzes the production of 1 μg p-Nitrophenol per minute (U/mL).

Endo-1,4-*β*-D-glucanohydrolase (EG) activity was also determined by DNS (Hong et al., 2001). The enzyme reaction system contained 1% CMCNa, acetate buffer (pH 4.8), and sample. After incubation at 50°C for 30 min, added DNS for color reaction. The absorbance value was measured at the wavelength of 540 nm. One unit of enzymatic activity of EG was described as: 1 mL enzyme solution catalyzes the production of 1 μg reducing sugar per minute (U/mL).

The measurement method of *β*-1, 4-glucosidase (BGL) activity was similar to that of CBH. The enzyme reaction system contained acetate buffer (pH 4.8), 5 mM 4-Nitrophenyl-*β*-D-glucopyranoside (PNPG), and crude enzyme solution. After incubation at 50°C for 10 min, added 1 M Na_2_CO_3_ for color reaction. The absorbance value was measured at the wavelength of 410 nm. One unit of enzymatic activity of BGL was described as: 1 mL enzyme solution catalyzes the production of 1 μg p-Nitrophenol per minute (U/mL).

The determination of xylanase activity was similar to that of FPA. The standard curve of xylose was constructed. Enzyme reaction system contained diluted sample, 100 mg/mL xylan (Source: Corn cobs). After incubation at 37°C for 30 min, added DNS for color reaction. The absorbance value was measured at the wavelength of 540 nm. One unit of enzymatic activity of xylanase was described as: 1 mL enzyme solution catalyzes the production of 1 mmol xylose per minute (U/mL).

Lignin peroxidase (Li P) activity was determined by resveratrol (Tanaka et al., 2009) with some improvements. 3 mL reaction mixture containing tartrate acid buffer (250 mM, pH 4.5), 40 mmol/L resveratrol, crude enzyme solution, and 20 mmol/L hydrogen peroxide. After reaction for 2 min, the OD value was measured at the wavelength of 310 nm. One unit of enzymatic activity of Li P was described as: 1 L enzyme solution catalyzes the oxidation of 1 μmol resveratrol per minute (U/L).

Mn-dependent peroxidase (Mn P) activity was determined in a reaction system containing lactic acid buffer (0.2 M, pH 5.0), 0.4 M MnSO_4_, crude enzyme solution, and 20 mM H_2_O_2_ (Peng et al., 2014). The change of OD value within 3 min was measured at the wavelength of 240 nm. One unit of enzymatic activity of Mn P was described as: 1 L enzyme solution catalyzes the oxidation of 1 μmol MnSO4 per minute (U/L).

Laccase (Lac) activity was determined in a reaction system containing acetic acid buffer (pH 5.0), 1 mM 2, 2′-azino-bis (3-ethylbenzothiazoline-6-sulfonic acid) (ABTS), and crude enzyme solution (Srivastava et al., 2013). The change of OD value within 2 min was measured at the wavelength of 430 nm. One unit of enzymatic activity of Lac was described as: 1 L enzyme solution catalyzes 1 μmol ABTS per minute (U/L).

### 2.8 Separation and purification of gCDH

Due to the addition of histidine purification tags at both ends of the target gene, the target protein can be affinity adsorbed with nickel chloride or nickel sulfate in the Ni column, and the concentrated fermentation supernatant of *A. niger* C112-*gcdh* was separated and purified by using a Ni-NTA column (Sangon Biotech). The concentration and purification process was as follows: 80% saturated ammonium sulfate (dissolved in 20 times) was added to the fermentation supernatant of *A. niger* C112-*gcdh* in an ice bath, and the precipitate was taken by centrifugation at 4°C, 10,000 rpm, and 10 min after being stored at 4°C for 12 h. The precipitate was re-dissolved with appropriate amount of pH 7.5 PBS solution and the supernatant was centrifuged at 4°C, 6000 rpm, 5 min. The supernatant was dialyzed in pH 7.5 PBS solution at 4°C for 12 h. After dialysis, the target protein gCDH was purified by Ni-NTA column (which can be stored at-80°C). And target protein was detected by 12% SDS-PAGE (Table 3).

**Table 3:** Preparation of 12%SDS-PAGE.

Components	Separation Gel (mL)	Stacking Gel (mL)
Distilled water	3.4	2.0
30% Acr-Bis (29:1)	4.0	1.0
Gel Buffer A/B	2.5	3.0
10% APS	0.1	0.06
TMEMD	0.006	0.006

### 2.9 Preliminary study of the enzymatic properties of gCDH

Under the optimal reaction conditions at different temperatures (25°C to 60°C), the activity of gCDH enzyme in 3 min was measured with lactose as substrate (see method 2.6); under the optimal reaction temperature, the enzyme solution was measured in buffer solutions with pH from 3.0 to 8.0, and the gCDH enzyme activity with lactose as the substrate is determined within 3 min. Determination of temperature and pH stability: the enzyme solution was incubated with pH 3–8 buffer at 4°C for 1 h and 20°C to 60°C for 5 h, respectively, and then the enzyme activity was determined.

## 3 Results

### 3.1 Cloning of CDH of *Grifola frondos*e

**Figure 1:**
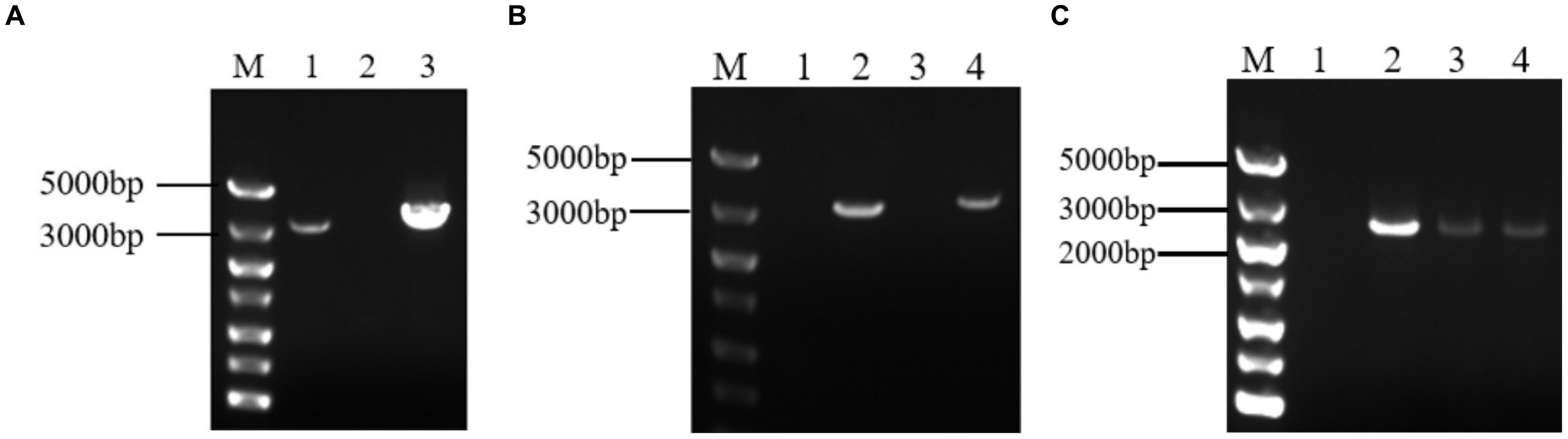
**(A)** Analysis of target gene PCR products by agarose gel electrophoresis. M: DNA maker DL5000; 1: positive control; 2: negative control; 3: PCR product of *gcdh*; **(B)** Agarose gel electrophoresis result of PCR of recombinant *A. niger* C112. M: DNA maker DL5000; 1: negative control; 2: positive control; 3: blank control of GF/GR; 4: *A. niger* C112-*gcdh*; **(C)** Agarose gel electrophoresis result of RT-PCR of recombinant *A. niger* C112. M: DNA maker DL5000; 1: blank control of GF/GR; 2–4: *A. niger* C112-*gcdh*.

The target fragment was obtained by electrophoresis of PCR products. And The fragment of 3,378 bp was *Grifola frondose* cellobiose dehydrogenase gene (*gcdh*) (Figure 1A). The target fragment was isolated and cloned into the pUCm-T vector, respectively, for sequencing. The sequencing results showed that *gcdh* contains 785 A, 902 T, 808 G, and 883 C, with G + C content of 50.06%. The CDH sequence of *Grifola frondosa* has 100% homology with the published CDH sequence of *Grifola frondosa* (AB083245.1) in NCBI.

### 3.2 gCDH amino acid sequence analysis, structure and property prediction

**Figure 2:**
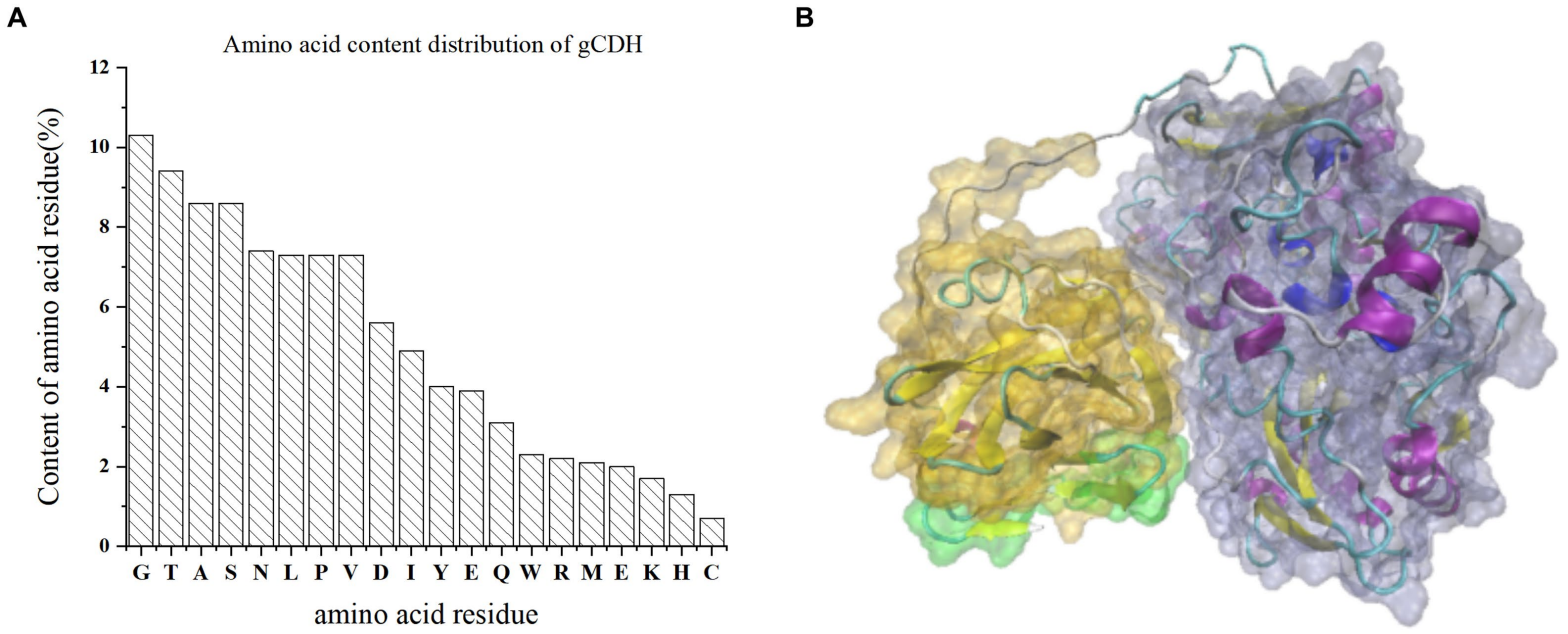
**(A)** Amino acid residue content distribution of gCDH; **(B)** Tertiary structure of gCDH; the green part is the signal peptide domain; the gray part is FAD-DH.

**Figure 3:**
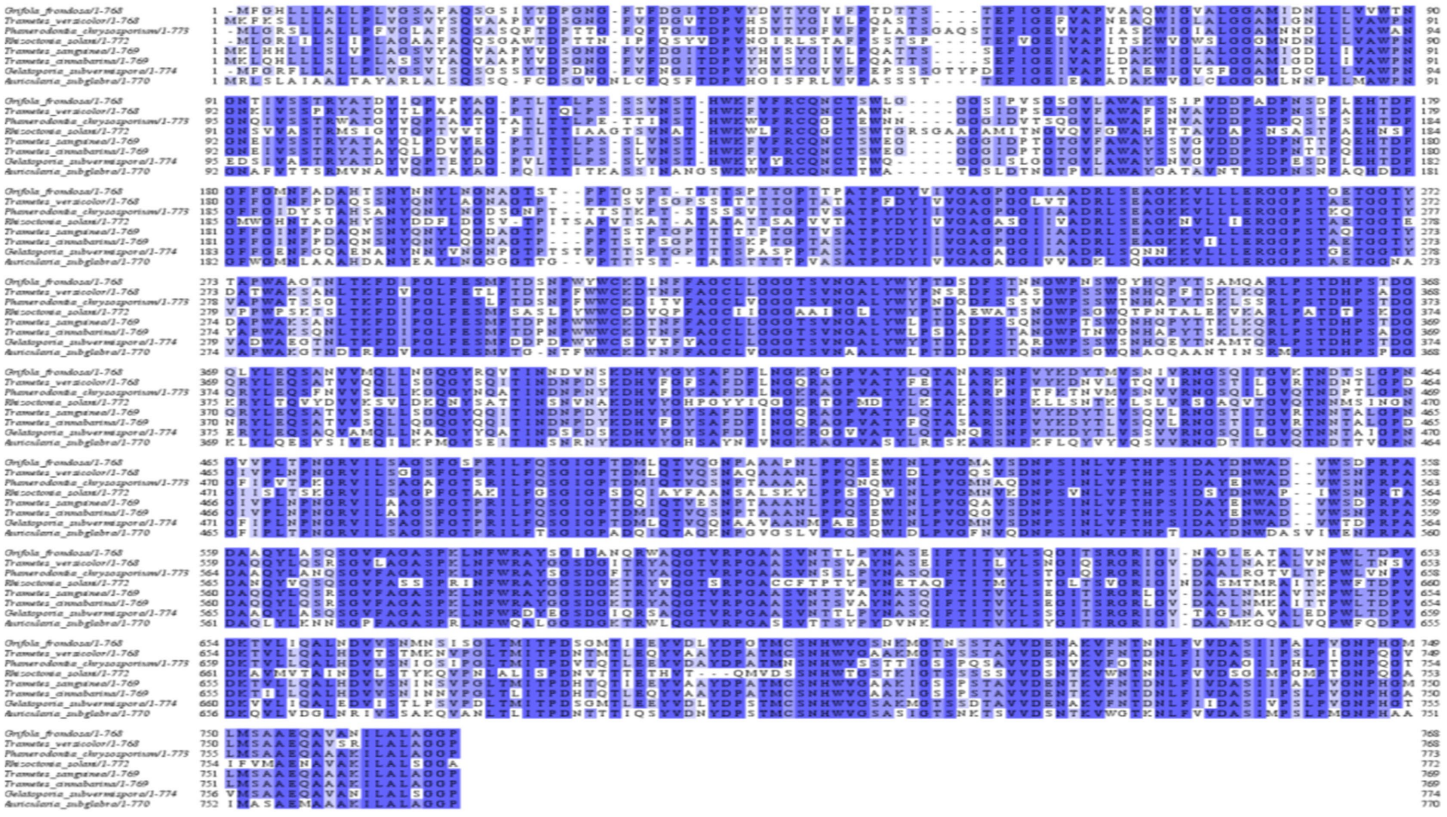
Multiple sequence analysis of CDH protein.

**Figure 4:**
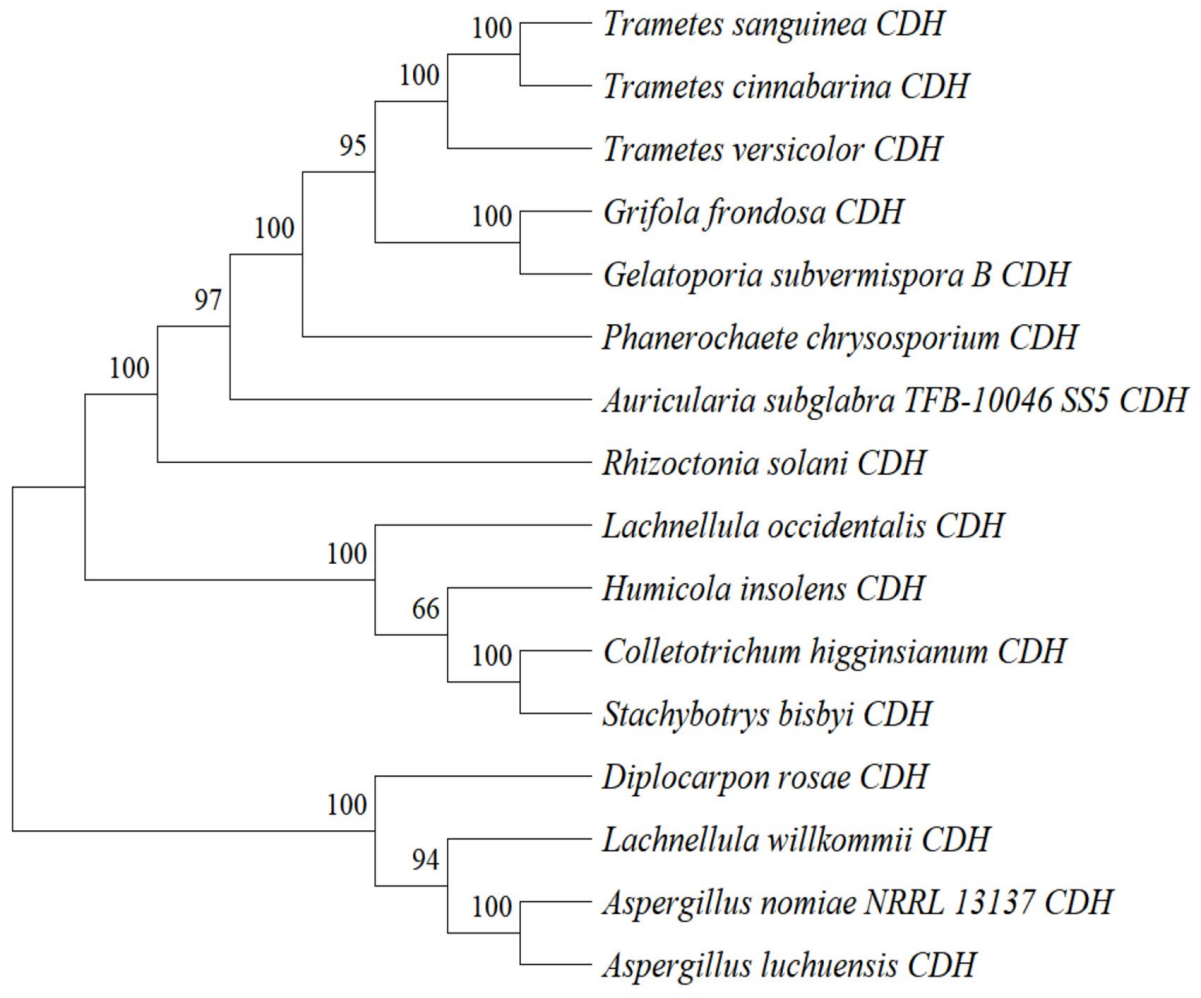
Phylogenetic tree analysis of amino acid sequences of cellobiose dehydrogenase from different sources.

By analyzing the amino acid sequence of gCDH, it has 768 amino acid residues, an isoelectric point of 4.5, a molecular mass of 81.5 KDa, a total of 58 negatively charged amino acid residues, and a total of 30 positively charged amino acid residues (Table 4). gCDH is a hydrophilic protein, with stable physical properties, and is an acidic protein. The protein secondary structure of gCDH was predicted through an online website, which showed that *ɑ*-helix accounted for 17.32%, *β*-folding accounted for 27.60%, and random coiling accounted for 55.08% of its secondary structure (Table 5). gCDH’s structural domains are shown in Table 6. The tertiary structure model of gCDH was predicted by homology modeling of known proteins in the protein database using SWISS-MODEL, an automated comparative protein modeling server, as shown in Figure 2. According to Jalview’s analysis of CDH protein conserved sequences, as shown in Figure 3, gCDH shares similar protein conserved sequences with other species. The phylogenetic analysis of CDH protein is shown in Figure 4, and the self unfolding value at the node is generally greater than 70, indicating that the phylogenetic tree is relatively reliable.

**Table 4:** Analysis results of amino acid sequences of gCDH.

Item	gCDH
The number of amino acids	768
Molecular weight	81467.81
Theoretical pI	4.5
Total number of negatively charged residues	58
Total number of positively charged residues	30
The number of C	3,643
The number of H	5,515
The number of N	951
The number of O	1,135
The number of S	21
Instability index	25.54
Aliphatic index	77.47
GRAVY	−0.081

**Table 5:** The distribution of secondary structure of gCDH.

Item	Content of secondary structure (%)
Random coil	55.08
Extended strand	27.60
Alpha helix	17.32

**Table 6:** Domains distribution of gCDH.

Domains	Range of amino acid residues
Signal peptide	1 to 18
CYT	24 to 193
FAD-DH	230 to 766

### 3.3 Transformation and expression of *Aspergillus niger C112-gcdh*

**Figure 5:**
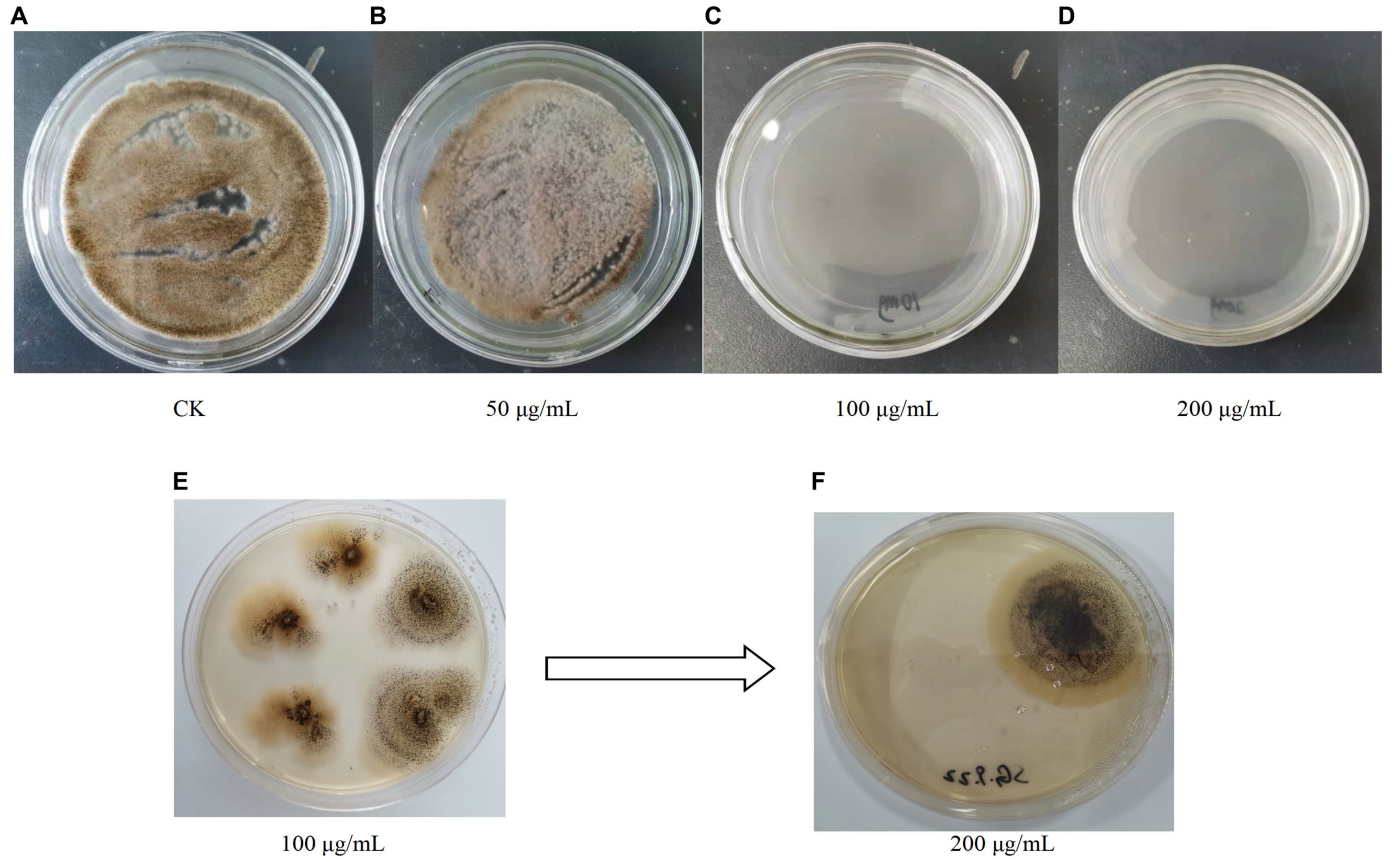
**(A–D)** Growth of *A. niger* C112 in different concentrations of hygromycin B; **(E,F)** Growth of recombinant *A. niger* C112 in different concentrations of hygromycin B.

After 200 μg/mL hygromycin B screening, the regenerated colonies were initially identified as positive transformants (Figure 5). The PCR products of the transformants were analyzed by agarose gel electrophoresis and the results were shown in Figure 1B. The bands of channel 2 were more than 3,000 bp, which was consistent with the size of *gcdh*. The result of RT-PCR products were shown in Figure 1C. The result showed that *gcdh* was successfully expressed in *A. niger* C112.

### 3.4 Activity of CDH

CDH activity was shown in Table 7. The CDH activity of *A. niger* C112 and *A. niger* C112-*gcdh* were 3.92 U/L, 36.63 U/L. The difference between the *A. niger* C112-*gcdh* group and the *A. niger* C112 group was very significant (*p* < 0.001). *A. niger* C112 itself have poor CDH activity, and the introduction of *gcdh* increased its CDH activity by 32.71 U.

**Table 7:** Enzyme activity estimator and diversity index.

Enzyme	*A. niger* C112	*A. niger* C112-*gcdh*	F^a^	P^b^
CDH	3.92 ± 1.59	36.63 ± 2.33	402.552	0.000
Cellulase	8.89 ± 0.63	11.43 ± 0.62	24.591	0.008
EG	19.84 ± 1.64	19.56 ± 2.80	0.22	0.889
CBH	2.35 ± 0.19	3.12 ± 0.43	7.890	0.48
BGL	40.03 ± 2.79	54.07 ± 3.65	28.013	0.006
Xylanase	0.84 ± 0.12	0.92 ± 0.11	0.792	0.424
Li P	1.51 ± 1.25	1.77 ± 0.97	0.087	0.783
Lac	0.24 ± 0.15	0.33 ± 0.15	0.645	0.467
Mn P	10.05 ± 0.92	22.28 ± 1.92	99.570	0.001

a *p* < 0.05 meaned the values of *A. niger* C112 group and *A. niger* C112-*gcdh* group were significantly different, *p* < 0.01 meaned the values of *A. niger* C112 group and *A. niger* C112-*gcdh* group were highly significant difference.

b Under the condition that there was a significant difference between *A. niger* C112 group and *A. niger* C112-*gcdh*, it indicated the effect of CDH on enzyme activity. The greater the *F* value, the greater the effect.

### 3.5 Analysis of the activity of cellulases

**Figure 6:**
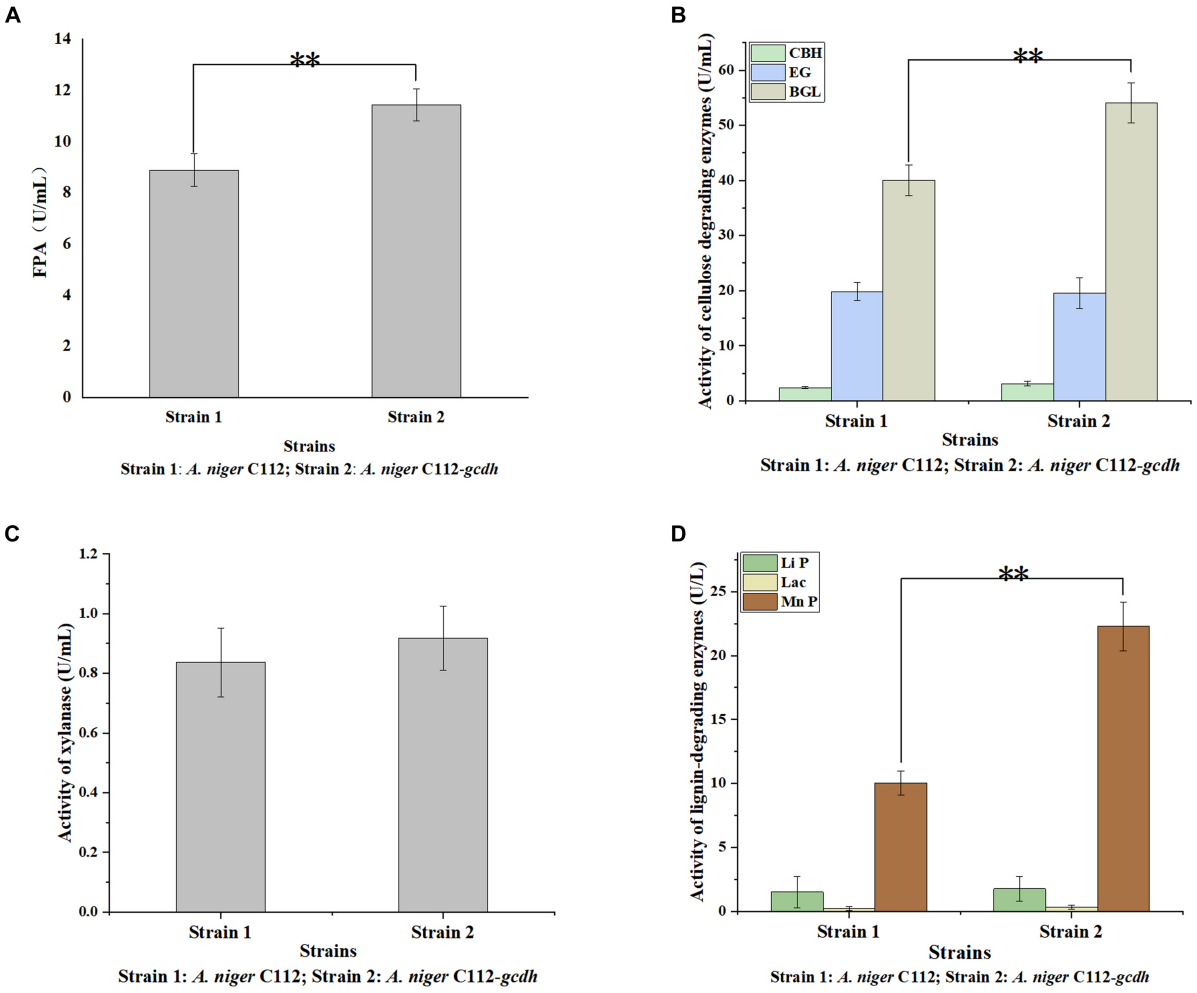
Activity of lignocellulose degrading enzymes of original *A. niger* and the recombinant *A. niger* strain. Cellulase activity was represented by FPA **(A)**; cellulase consists of EG, BGL, and CBH **(B)**; Hemicellulose degrading enzyme was mainly xylanase **(C)**; lignin-degrading enzymes were mainly Li P, Lac, and Mn P **(D)**; *p < 0.05 vs. *A. niger* C112; **p < 0.01 vs. *A. niger* C112; ***p < 0.001 vs. *A. niger* C112.

Cellulases is the main cellulose-degrading enzyme. Cellulases activity was represented by filter paper enzyme activity (FPA), which was shown in Figure 6A. The FPA of *A. niger* C112 and *A. niger* C112-*gcdh* were 8.89 U/mL and 11.43 U/mL relatively. There was a highly significant difference in FPA of the two strains (*p* < 0.01). Phillips et al.’s (2011) study showed that adding CDH to the fermentation system of *cdh* deficient strain can increase cellulases activity. In this study, the cellulases activity of *A. niger* C112-*gcdh* was 28.57% higher than that of *A. niger* C112 cellulases activity.

To further clarify the function of CDH on cellulases, we determined the activity of EG, BGL, and CBH which were shown in Figure 6B. The EG activity of *A. niger* C112 and *A. niger* C112-*gcdh* were 19.84 U/mL and 19.56 U/mL. The BGL activity of *A. niger* C112 and *A. niger* C112-*gcdh* were 40.03 U/mL and 54.07 U/mL. The CBH activity of *A. niger* C112 and *A. niger* C112-*gcdh* were 2.35 U/mL and 3.12 U/mL. There was no significant difference in the activity of EG and CBH of the two strains (*p* > 0.05). However, there was a highly significant difference in BGL enzyme activity between *A. niger* C112 and group *A. niger* C112-*gcdh* (*p* < 0.01). The BGL activity of *A. niger* C112-*gcdh* was increased 35.07% relatively, compared with the original strain *A. niger* C112. The result showed that *gcdh* enhanced the activity of BGL, but had no significant effect on the activity of EG and CBH.

### 3.6 Analysis of the activity of xylanase

Hemicellulose degrading enzymes include xylanase, xylosidase, mannanase, and other enzymes and *A. niger* had a relatively complete xylanase system (Pel et al., 2007). Xylanase activity was shown in Figure 6C. The xylanase activity of *A. niger* C112 and *A. niger* C112-*gcdh* were 0.84 U/mL and 0.92 U/mL relatively. There was no significant difference in the activity of xylanase of the two strains (*p* > 0.05). The result showed that *gcdh* had no significant effect on the xylanase activity of *A. niger* C112. And no research showed that CDH will affect xylanase activity.

### 3.7 Analysis lignin degrading enzymes

Lignin degrading enzymes mainly include Lignin peroxidase (Li P), Laccase (Lac), and Mn-dependent peroxidase (Mn P). The researches of dos Santos et al. (2016) confirmed that *Aspergillus* fungi had lignin degradation ability. The activity of Li P, Lac, and Mn P were shown in Figure 6D. The Li P activity of *A. niger* C112 and *A. niger* C112-*gcdh* were 1.51 U/mL and 1.77 U/L. The Lac activity of *A. niger* C112 and *A. niger* C112-*gcdh* were 0.24 U/L and 0.33 U/L. The Mn P activity of *A. niger* C112 and *A. niger* C112-*gcdh* were 10.05 U/L and 22.28 U/L. There was no significant difference in the activity of Li P and Lac of the two strains (*p* > 0.05). But there was a significant difference in Mn P activity between *A. niger* C112 and *A. niger* C112-*gcdh* (*p* < 0.01). The Mn P activity of *A. niger* C112-*gcdh* was increased 121.69%, compared with the original strain *A. niger* C112. The result showed that *gcdh* enhanced the activity of Mn P, but had no significant effect on the activity of Li P and Lac. Significance analysis of all enzyme activity was shown in Table 7, which showed that the activity of CDH, cellulase, BGL, and Mn P in *A. niger* C112-*gcdh* were significantly increased, and the effect of *cdh* on MnP was greater than that on cellulase and BGL.

### 3.8 Isolation and purification of gCDH

**Figure 7:**
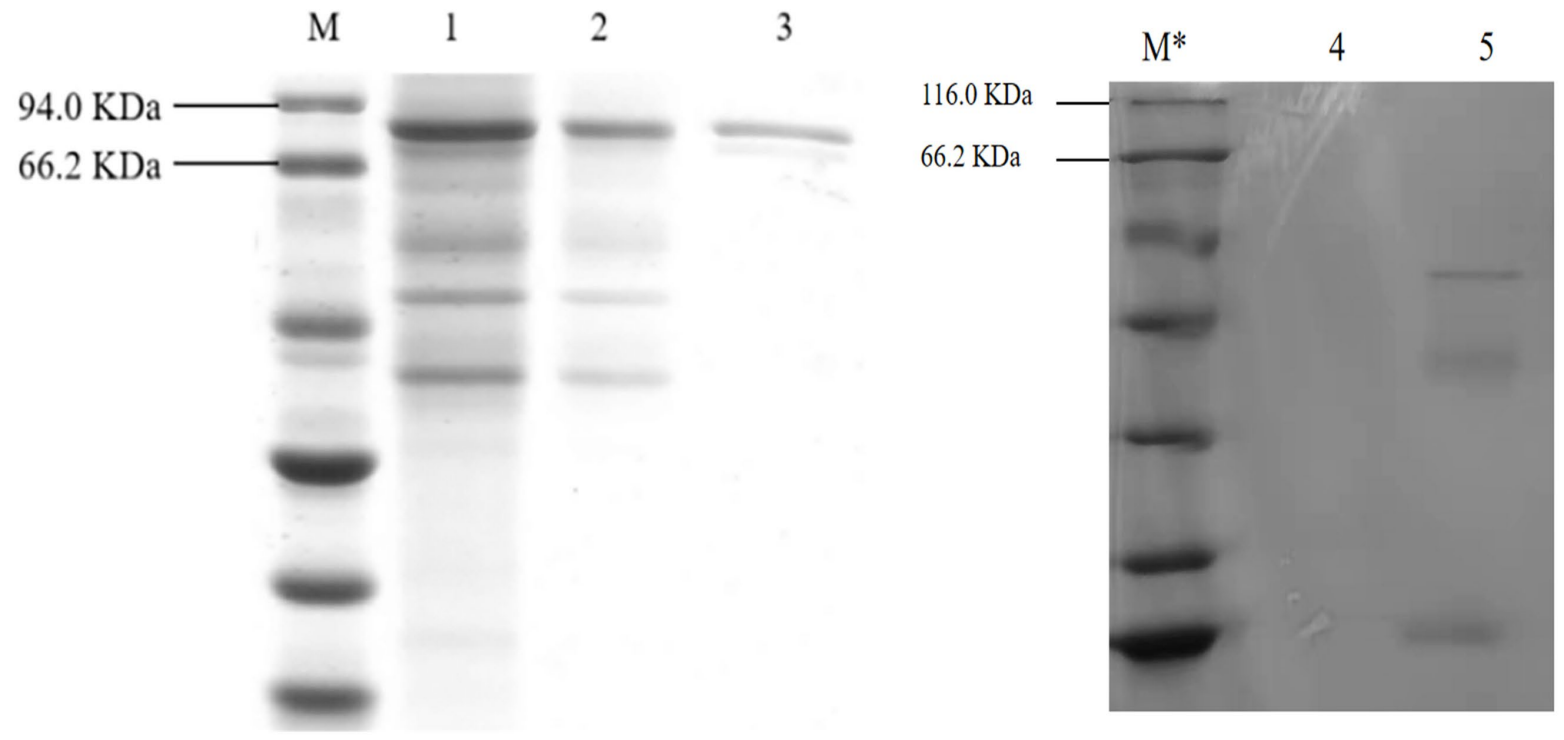
SDS-PAGE electrophoresis of *A. niger* C112 and recombinant *A. niger* C112 gCDH. M: marker 94.0 KDa; 1: recombinant *A. niger* C112 crude enzyme solution; 2: recombinant *A. niger* C112 heteroproteins; 3: recombinant *A. niger* C112 gCDH; M*: 116.0 KDa; 4: CK; 5: *A. niger* C112.

As shown in Figure 7, after the protein of *A. niger* C112-*gcdh* was isolated and purified, a protein band of around 80 KDa was obtained, which is consistent with the size of the molecular weight of gCDH protein predicted by the online website (ExPASy-ProtParam). However, *A. niger* C112 had almost no bands at the 80 KDa band, and the expression of CDH significantly increased after adding the *gcdh* gene.

### 3.9 Analysis of the enzymatic properties

**Figure 8:**
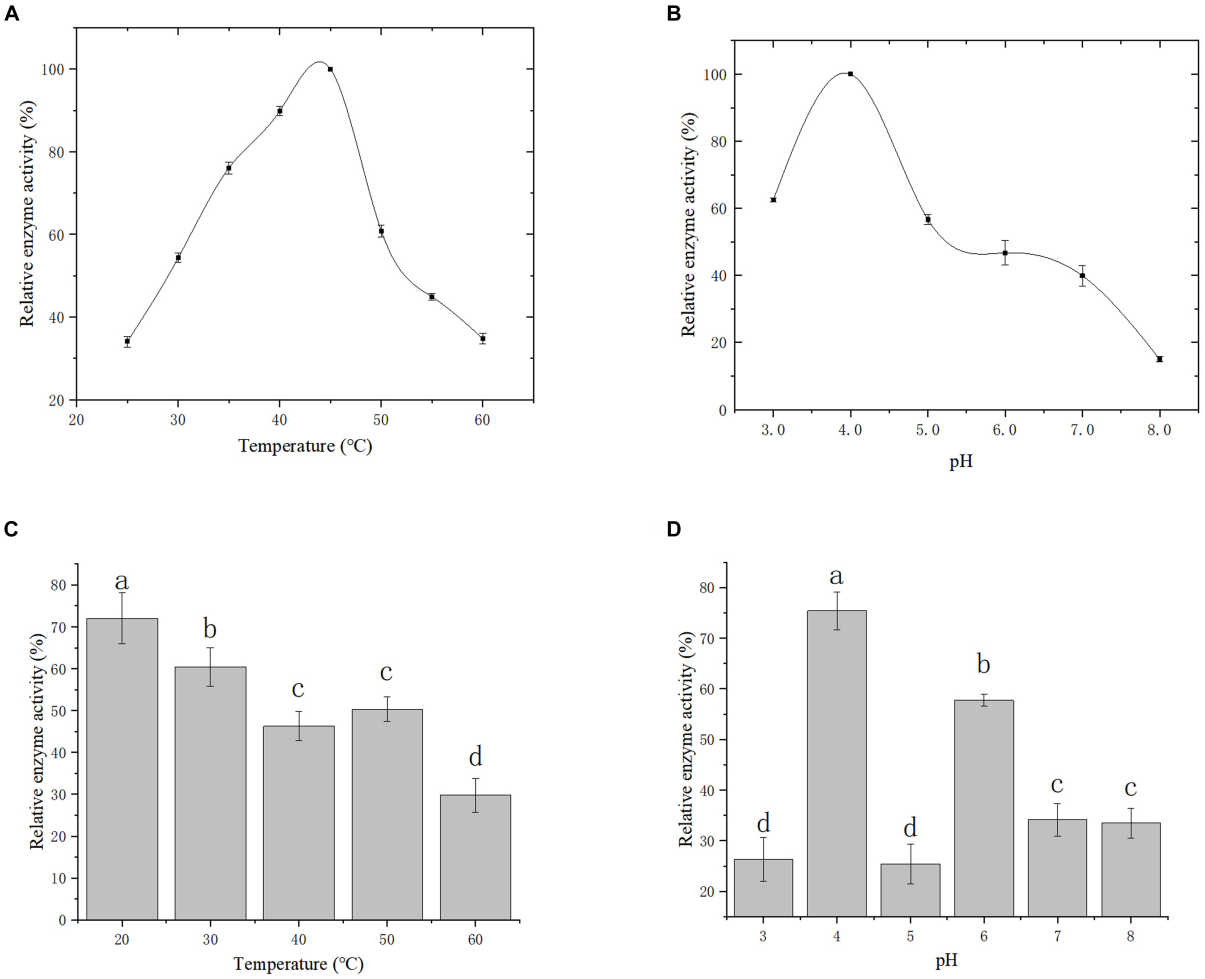
Effect of temperature **(A)** and its stability **(B)** on recombinant CDH; Effect of pH **(C)** and its stability **(D)** on recombinant CDH.

Effect of temperature and pH on gCDH enzyme activity: The enzyme activity was measured under different temperature conditions as shown in Figure 8A to reflect the variations of enzyme activity as temperature altered. It was observed that the optimal temperature for gCDH is 45°C. Furthermore, the activity of gCDH in different pH (3.0–8.0) was determined at the optimal temperature, and the optimal pH was obtained at 4.0 (Figure 8B). Determination of temperature and pH stability: The enzyme was incubated in the range of 20°C to 60°C for 5 h and its enzyme activity remained above 70% at 20°C compared to the initial enzyme activity (Figure 8C). he enzyme was incubated in pH 3–8 buffer for 1 h and compared to the initial enzyme activity it was found that the enzyme activity could be maintained above 75% at pH 4 (Figure 8D).

## 4 Conclusion

In this study, the cellulase, exoglucanase, cellulases, and manganese peroxidase activities of the constructed *A. niger* C112-*gcdh* strain were significantly increased. Compared with *A. niger* C112, the activities of cellulases, exoglucanase, and manganese peroxidase were increased by 28.57, 32.77, 35.07, and 121.69%, respectively. It was proved that recombinant gCDH could improve the activity of cellulases, exoglucanase, *β*-1, 4-glucosidas and manganese peroxidase produced by *A. niger*. Research has shown that the gCDH produced by *A. niger* C112-*gcdh* has potential in the degradation of lignocellulose. The optimal pH for gCDH is 4, which is consistent with PredictProtein’s prediction that it is an acidic protein. Within the measurement range, the stability of gCDH temperature and pH can both be above 70%, and the physical properties are relatively stable. However, the CDH enzyme activity in this study is relatively low compared to other studies, and further exploration is needed to determine the conditions for the enzyme production medium of *A. niger* C112-*gcdh*.

## 5 Discussion

In addition to its important role in lignocellulosic interpretation, CDH can also be applied in various fields such as ecological remediation, antimicrobial function, biofuel cells and biosensors (Wu et al., 2021). Many studies have focused on the enzymatic properties of CDH, but there are still few studies on the effect of CDH on lignocellulose degrading enzymes. And most of the studies on the effect of CDH on lignocellulose degrading enzymes are independent of the organism, which is also because the enzymes secreted by the organism are very complex, which brings difficulties to the research. In this study, we did some small work on the interaction between CDH and lignocellulose degrading enzymes secreted by fungi. We have constructed a CDH-engineered strain *A. niger* C112-*gcdh*, have achieved normal expression of *gcdh* in *A. niger* C112, and have isolated and purified gCDH. In addition, the optimal temperature of gCDH was determined to be 45°C, and the optimal PH was 4.0. CDH from various sources had been successfully expressed in *Pichia pastoris*, *Trichoderma reesei*, and *A. niger* (Ma et al., 2017; Banerjee and Roy, 2021). Ma et al. (2017) and Bey et al. (2011) expanded the scale of fermentation and made the activity of CDH reach a relatively high level. Other scholars obtained high CDH activity by solid medium fermentation. Compared with the researches of other scholars, CDH activity in this article was lower at 36.63 U/L, probably because the culture conditions used were more suitable for the production of cellulase rather than CDH. The culture methods and conditions can be further optimized. The low activity of the lignin-degrading enzyme of *A. niger* C112 might be that alkali-treated poplar mainly removes the lignin of poplar fiber, resulting in the reduction of lignin content, lacking inducers. Huang et al.’s (2001) study proved that CDH can enhance lignin degradation by Mn P. In this paper, the Mn P activity of *A. niger* C112 after introduction into *gcdh* was 2.22 times higher than before. In addition, CDH may interact with many other enzymes that we do not know, after all, *A. niger* has a complex secretory system, which works together to degrade lignocellulose, clarifying their mechanism of action will be the next research plan.

In the global energy crisis, the development and utilization of biomass energy are important methods to solve the problem. Based on previous studies and this study, we can see the potential of CDH in biomass conversion. Introducing the *gcdh* gene into *A. niger* C112 not only improves its own CDH activity but also enhances the activity of cellulases, which leads to a more efficient biomass conversion. Constructing strains containing *gcdh* gene gives us a solution to realize the high efficiency of biomass conversion, and more scholars need to invest in the research.

## Data availability statement

The original contributions presented in the study are included in the article/Supplementary material, further inquiries can be directed to the corresponding author.

## Author contributions

YZ: Writing – original draft, Conceptualization, Data curation, Formal analysis, Software. ZG: Conceptualization, Data curation, Formal analysis, Writing – original draft, Writing – review & editing. ML: Funding acquisition, Project administration, Writing – review & editing. XJ: Supervision, Writing – review & editing. BZ: Conceptualization, Funding acquisition, Resources, Supervision, Writing – review & editing.
