# Supplementary material for: Expression of cellobiose dehydrogenase gene in Aspergillus niger C112 and its effect on lignocellulose degrading enzymes
Source: Front Microbiol. 2024 Mar 18;15:1330079. doi: 10.3389/fmicb.2024.1330079 (PMC10982475; doi:10.3389/fmicb.2024.1330079)
Supplement: Supplementary file 1 [file Data_Sheet_1.PDF]

Supplementary Figures

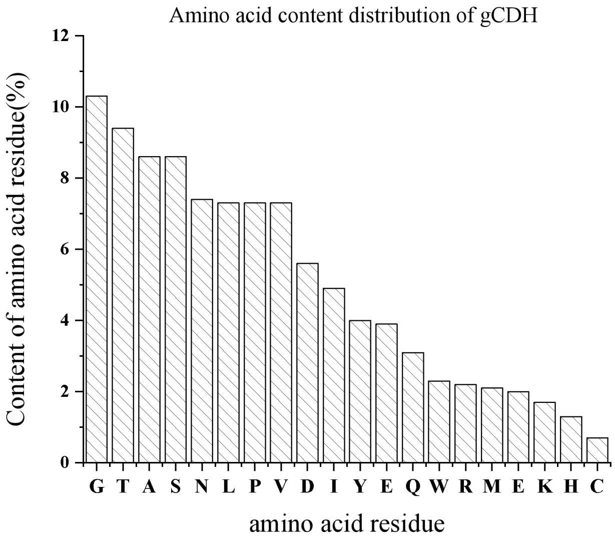

Supplementary Figure 1. Amino acid residue content distribution of gCDH

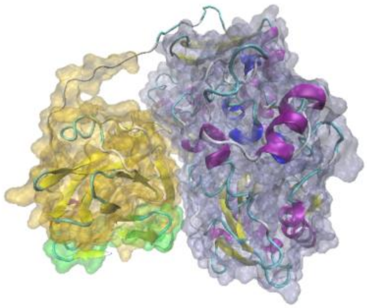

Supplementary Figure 2. Tertiary structure of gCDH.The green part is the signal peptide domain; The yellow part is CYT; The gray part is FAD-DH

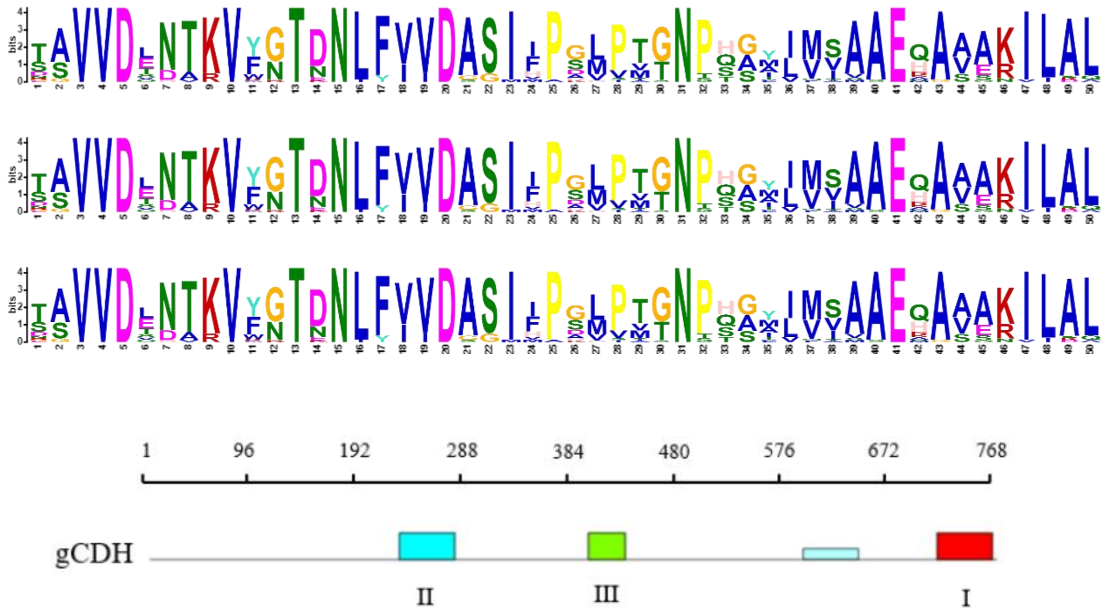

Supplementary Figure 1. The top three motifs obtained from the alignment of 30 CDH amino acid sequences and their locations on gCDH. From top to bottom were Ⅰ, Ⅱ, and Ⅲ.

Supplementary Tables

Supplementary Table 1. Analysis results of amino acid sequences of gCDH

Item	gCDH
The number of amino acids	768
Molecular weight	81467.81
Theoretical pI	4.5
Total number of negatively charged residues	58
Total number of positively charged residues	30
The number of C	3643
The number of H	5515
The number of N	951
The number of O	1135
The number of S	21
Instability index	25.54
Aliphatic index	77.47
GRAVY	-0.081

Supplementary Table 2. The distribution of secondary structure of gCDH

Item	Content of secondary structure (%)
Random coil	55.08
Extended strand	27.60
Alpha helix	17.32

Supplementary Table 3. Domains distribution of gCDH

Domains	Range of amino acid residues
Signal peptide	1 to 18
CYT	24 to 193
FAD-DH	230 to 766

Supplementary Table 4. Source of CDH amino acid sequences involved in comparison

Filamentous fungi	Accession number*	Filamentous fungi	Accession number*
Trametes versicolor	AAC50004.1	*Humicola insolens*	AAF69005.1
Grifola frondose	BAC20641.1	*Irpex lacteus*	BAD36748.1
Volvariella volvacea	ATJ01065.1	*Neurospora crassa*	XP_956591.1
Auricularia subglabra	EJD48894.1	*Phlebia lindtneri*	AGE97206.1
Athelia rolfsii	AAO64483.1	*Phanerochaete chrysosporium*	AAC49277.1
Aspergillus nomiae	XP_015405088.1	*Rhizoctonia solani*	CUA69410.1
Aspergillus luchuensis	GAT21023.1	*Schizophyllum commune*	XP_003026061.1
Crassicarpon hotsonii	ABS45567.2	*Stachybotrys bisbyi*	ADT70778.1
Coniophora puteana	EIW75667.1	*Trametes sanguinea*	AGS09132.1
Colletotrichum orbiculare	TDZ26016.1	*Trametes cinnabarina*	AAC32197.1
Colletotrichum higginsianum	TIC92379.1	*Thermothelomyces thermophilus*	AAC26221.1
Cerrena unicolor	AGS09133.1	*Termitomyces clypeatus*	AOW70087.1
Diplocarpon rosae	PBP22038.1	*Lachnellula occidentalis*	TVY48802.1
Gelatoporia subvermispora	EMD36613.1	*Lachnellula willkommii*	TVY91786.1
Hypoxylon haematostroma	ADT70775.1	*Talaromyces islandicus*	CRG83596.1

*All amino acid sequences were derived from NCBI (Home - Protein - NCBI (nih.gov).
