# Supplementary material for: Expression of cellobiose dehydrogenase gene in Aspergillus niger C112 and its effect on lignocellulose degrading enzymes
Source: Front Microbiol. 2024 Mar 18;15:1330079. doi: 10.3389/fmicb.2024.1330079 (PMC10982475; doi:10.3389/fmicb.2024.1330079)
Supplement: Supplementary file 2 [file Data_Sheet_2.PDF]

Supplementary Material

pH 5.5 sterile osmotic stabilizer: 5 mM K2HPO4, 5 mM KH2PO4, 0.8 M MgSO4

pH 7.5 sterile isotonic solution: 10 mM Tris-HCl, 50 mM CaCl2, 1.2 M D-sorbitol

1 mL/L trace elements : 1 g/L FeSO4•7H2O, 8.8 g/L ZnSO4•7H2O, 0.4 g/L CuSO4•5H2O, 0.15 g/L MnSO4•4H2O, 0.1 g/L Na2B4O7•10H2O, 50 mg/L (NH4)6Mo7O24•4H2O, 0.2 mL/L HCl

pH 7.5 mediated solution: 50%(W/V) PEG-4000, 1 mM CaCl2, 10 mM Tris-HCl

The top regeneration medium contains: 0.6 g/L $NaNO_3$, 3.4 g/L CsCl, 0.52 g/L KCl, 1,52 g/L KH_2PO_4, 1.2 M D-sorbitol, 1 mL/L trace elements, 10 g/L glucose, 0.5 g/L $MgSO_4$, and 0.8% agar

A. niger solid medium: 10 g/L peptone, 0.5 g/L $CaCl_2$, 4.0 g/L KH_2PO_4, 1 mL/L trace elements, 10 g/L glucose, 1.0 g/L $MgSO_4$, and 2 % agar

Fermentation medium: 50 g/L pretreated poplar powder, 10 g/L peptones, 4 g/L KH_2PO_4, 1.0 g/L $MgSO_4$, 0.5 g/L $CaCl_2$, 0.04% tween-80, and 1 mL/L trace elements
