# Supplementary figures and images for: Expression of cellobiose dehydrogenase gene in Aspergillus niger C112 and its effect on lignocellulose degrading enzymes
Source: Front Microbiol. 2024 Mar 18;15:1330079. doi: 10.3389/fmicb.2024.1330079 (PMC10982475; doi:10.3389/fmicb.2024.1330079)

## Slide 1
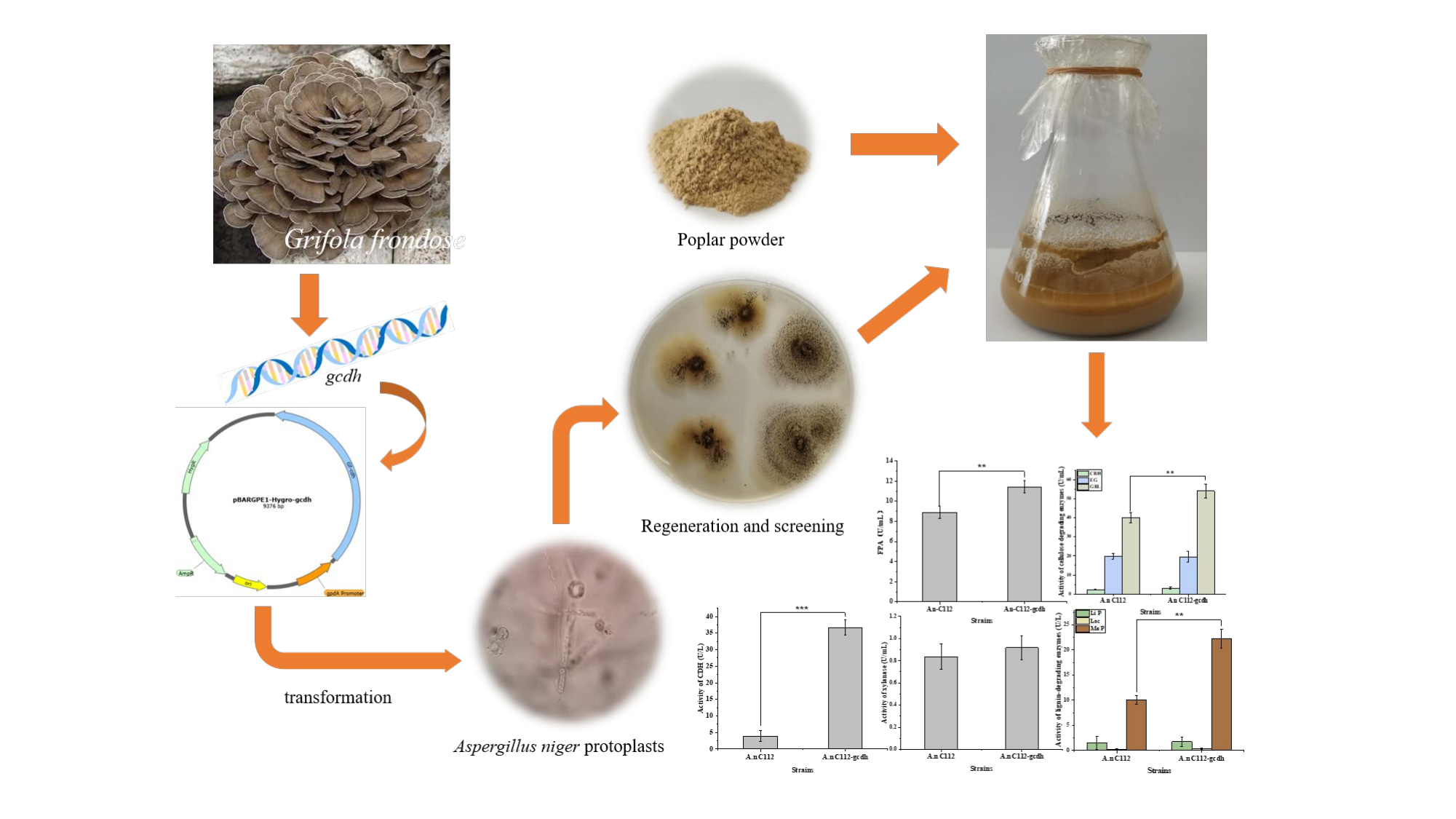

Supplement: Supplementary file 3 [file Presentation_1.PPTX]
